# Notes and Comments

**Published:** 1898-04

**Authors:** 


### Notes and Comments.[1]

Calomel as a Curative Agent in Diphtheria.[2]—Dr. Leonardo D. Judd, of Philadelphia, in the November, 1897, number of the *Annals of Hygiene and of Medicine*, recommends heroic doses of calomel in treating this disease. To an infant of eighteen months,

[1] The assistant editor solicits contributions for this department,—new methods, new remedies and formulas, or any short practical note which may prove of value to the practitioner or student. Address 1338 Walnut Street, Philadelphia.

[2] A paper read before the meeting of the American Climatological Association.

with malignant diphtheria which had steadily progressed towards a fatal issue in the face of the usual treatment as generally taught and employed, until she was in a nearly moribund condition, he gave ten grains of calomel in water at the first dose, and followed this with five grains every hour until a full, free, characteristic dejection occurred. It took eighty grains to reach such an effect; the child commenced to improve from the fourth dose, and made a splendid recovery; not the slightest ill effect followed. In another case, an adult, he began with a scruple at the first dose, and ten grains every hour thereafter for thirty-five hours, when a copious dejection occurred; smaller ones had preceded, but not until three hundred and sixty-five grains had been given did he get the characteristic action. He uses it also in some cases, in solution, to syringe the nostrils, etc. He regards it as having diagnostic value, having found that where this disease exists the usual action of calomel is resisted until the disease itself is overcome, and says, "Believing, as I do, that all sore throats, where the tonsils are involved and where false membrane exists, are in some degree related to diphtheria, I never hesitate to exhibit calomel in small doses at first, and by its action prove or disprove the diphtheritic tendency of the case. If the tenth of a grain given every hour, dry, upon the tongue, fails to act upon the bowels in any reasonable time,—say, in from eight to twelve hours,—I assume that there is more than an ordinary condition existing, and strengthen the dose or shorten the time between the doses accordingly." He further says, "In true diphtheria calomel is slow to act upon the bowels, and herein lies its diagnostic value, if in doubt. The more severe the type the greater the quantity to be given. I have spoken of the *characteristic dejection* to be secured, especially in the malignant type, before we should let up in the administration of the drug. It should be a green, copious, frothy discharge, resembling 'frog spittle,' such as may be seen in an old water-trough. There is no danger of ptyalism. So long as it has the bacilli to antagonize, that alone seems to demand all its energy; and when it has conquered, it leaves in the characteristic evacuation described, and the patient is *absolutely free from any mal-effect.* I have never yet seen a case of diphtheria salivated by calomel in large or small doses, nor have I seen it create hypercatharsis in any case of diphtheria."

---

GEOGRAPHY OF THE TEETH.—Referring to the interesting series of articles published in the *Popular Science Monthly* on the racial geography of Europe, the *Dominion Dental Journal* says, editorially, "During a prolonged pedestrian tour in Brittany and Normandy, we were much struck with the difference in the physical types in favor of the latter. Normandy contains the blondest people of France, Brittany the darkest and most benighted. Even the cattle have marked differences which, with the contrasts in the human types, are attributed to the influences of physical environment. Progress and prosperity show their effects in superior physique. Normandy and good teeth; Brittany and bad teeth. Brittany is the most devout (superstitious). The native Breton peasants are the filthiest people in Europe."

---

Gelatine Substitute for Glass.—The *Scientific American*, in commenting upon the new substitute for glass, called tectorium, says it has been found satisfactory in Germany for the following reasons: (1) It can be bent without being broken; (2) is both tough and flexible; (3) is not softened by the rays of the sun; (4) is non-soluble; (5) is not affected by severe cold; (6) is a bad conductor of heat; (7) is well adapted for roofs, on account of its extreme lightness; (8) when exposed to the sun it loses its original yellowish color in time and becomes harder and more durable; (9) can be made, by a very cheap process, to imitate stained glass in such manner that it cannot be distinguished from the genuine article; (10) can be cut by shears, nailed to wood, and transported without danger; (11) can be easily repaired in case it is cut; (12) does not break; and (13) is well adapted for factory windows and skylights for hot-houses, market halls, verandas, transportable buildings, and for roofing. It is further stated that it has as yet only been sold in small quantities and in a few places, the product still being an experiment.

---

New Means for arresting Hemorrhage.—The matter of arresting hemorrhage, even from slight wounds, sometimes is a serious problem. A novel treatment was recently adopted by Dr. Bienwald, and reported in the *American Journal of Surgery*. In the treatment of a small child, the subject of hæmophilia, having failed to arrest the hemorrhage from a small wound on the face by the application of perchloride of iron, he obtained some blood by aspiration from a healthy subject and deposited it on the wound. In a few minutes it coagulated, and the hemorrhage at once ceased. His explanation of the action of the remedy is that it supplies the ferment necessary for thrombosis in the small vessels. Whether this is correct or not it is impossible to say in the absence of definite knowledge of the pathology of hæmophilia. As affording his explanation some support may be mentioned the success obtained by Wright in his experiments with a solution of fibrin ferment and chloride of calcium as a styptic. Bienwald's ingenious method certainly deserves careful investigation.

---

TREATMENT OF ROOT-PERFORATION.—Dr. Register advises, in the treatment of root-perforation, to pack in the canal and against the pericementum, at the point of perforation, a small quantity of salol, and over this place a cone of zinc phosphate. He adds, "Of course, the salol disappears, as it always does after a period when used as a canal filling, but while it lasts it performs its office as an unirritating antiseptic." From our clinical experience, we would say, the fact that the salol disappears is reason sufficient to discard it, either for the purpose spoken of or for a root-canal filling. For closing these perforations there is nothing better, after thorough sterilization, than a small quantity of thick chloro-percha.

---

THE DENTIST AND MORALS.—A recent medical writer says, "A physician who cannot assimilate morals into his being can never expect to amount to much as a man. In other words, a physician as a man is measured by the depth of his moral nature, and not by the pretensions to professional greatness with which he would veneer his real self." This is quite as true of the dentist. It is for this reason that many men seemingly great are really small. They are out of harmony with true success, because they are out of harmony with their better selves.

---

IMITATION GRANULAR GUM.—Dr. E. A. Randall, in the *Dominion Dental Journal*, gives the following method for imitating the granular appearance of the natural gum: "When using plain teeth and pink rubber, instead of finishing gum with file and sand-paper, use with the dental engine a large round bur (rather dull); a smaller bur in the corners between the teeth. With the rapidly revolving bur carve the gum festoons, moving first vertically and then longitudinally; as the carving process nears completion pass the bur lightly over the surface, then polish with brush-wheels, pumice, and whiting. This gives that granular appearance peculiar to the natural gum, and not a perfectly smooth surface."

---

Hot Water to relieve Pain.—In a paper read before the New Jersey State Dental Society, Dr. E. H. Allen advised the following treatment:

"To reduce pain and inflammation of the root of the tooth in cases of pericementitis, also the pain occasioned by a setting of a crown or bridge, apply hot water to the gums and about the root of tooth or teeth affected. For the application of the hot water, use a two-quart fountain syringe, hung about six feet from the floor, conduct the water through rubber tubing to the mouth of the patient, delivered through a nozzle with an opening about one-twenty-fourth of an inch; water to be as hot as can be borne in the mouth. The water can be taken out by the saliva-ejector attached to the fountain spittoon. This will in most cases give quick relief."

Our method of treating, under similar conditions, is to have the patient hold hot water in the mouth, which is repeated several times. This assists the capillaries in establishing a more normal circulation, thus relieving the pain.

